# Brain functional connectivity and network characteristics changes after vagus nerve stimulation in patients with refractory epilepsy

**DOI:** 10.1515/tnsci-2022-0308

**Published:** 2023-09-07

**Authors:** Yongqiang Ding, Kunlin Guo, Xinjun Wang, Mingming Chen, Xinxiao Li, Yuehui Wu

**Affiliations:** Department of Neurosurgery, The Fifth Affiliated Hospital of Zhengzhou University, Zhengzhou, China; Henan Key Laboratory of Brain Science and Brain–Computer Interface Technology, School of Electrical and Information Engineering, Zhengzhou University, Zhengzhou, China

**Keywords:** vagus nerve stimulation, refractory epilepsy, graph theory, functional connectivity, small-world metrics, EEG

## Abstract

**Objective:**

This study aims to investigate the impact of vagus nerve stimulation (VNS) on the connectivity and small-world metrics of brain functional networks during seizure periods.

**Methods:**

Ten refractory epilepsy patients underwent video encephalographic monitoring before and after VNS treatment. The 2-min electroencephalogram segment containing the ictal was selected for each participant, resulting in a total of 20 min of seizure data. The weighted phase lag index (wPLI) and small-world metrics were calculated for the whole frequency band and different frequency bands (delta, theta, alpha, beta, and gamma). Finally, the relevant metrics were statistically analyzed, and the false discovery rate was used to correct for differences after multiple comparisons.

**Results:**

In the whole band, the wPLI was notably enhanced, and the network metrics, including degree (D), clustering coefficient (CC), and global efficiency (GE), increased, while characteristic path length (CPL) decreased (*P* < 0.01). In different frequency bands, the wPLI between the parieto-occipital and frontal regions was significantly strengthened in the delta and beta bands, while the wPLI within the frontal region and between the frontal and parieto-occipital regions were significantly reduced in the beta and gamma bands (*P* < 0.01). In the low-frequency band (<13 Hz), the small-world metrics demonstrated significantly increased CC, D, and GE, with a significantly decreased CPL, indicating a more efficient network organization. In contrast, in the gamma band, the GE decreased, and the CPL increased, suggesting a shift toward less efficient network organization.

**Conclusion:**

VNS treatment can significantly change the wPLI and small-world metrics. These findings contribute to a deeper understanding of the impact of VNS therapy on brain networks and provide objective indicators for evaluating the efficacy of VNS.

## 1 Introduction

Epilepsy is one of the most common central nervous system disorders and nearly 70 million people have been diagnosed with epilepsy in the world [1]. Most epileptic patients can control their seizures by taking antiseizure medications (ASMs). However, 30% of the patients cannot effectively control their seizures after combined treatment with a variety of ASM and developed refractory epilepsy (RE) [2]. At present, for patients with RE, it is still the first choice to control seizures by excising the epileptic focus [3]. However, in some patients, the epileptic focus cannot be accurately located or is located in important functional areas. These should not be removed, which could lead to serious postoperative complications (motor dysfunction, visual field defect, etc.) [4]. Neuromodulation, which involves stimulating brain regions with a weak electrical current, can control seizures without damaging brain tissue [5]. Therefore, neuromodulation is the primary treatment for patients who cannot undergo surgical resection [6].

Vagus nerve stimulation (VNS) has been used to treat RE for more than 30 years [7]. In a retrospective follow-up study, it was found that the frequency of focal epilepsy and generalized epilepsy was significantly reduced 1 year after VNS implantation compared with that before VNS treatment [8]. However, the exact mechanism by which VNS treats epilepsy remains unclear. The locus coeruleus-norepinephrine theory is one of the widely accepted mechanisms of VNS regulation [9]. Since the vagus nerve fibers project to the locus coeruleus (LC), which is the main source of the release of norepinephrine (NE), VNS can significantly affect the release of NE under different stimulation parameters. NE plays an important role in the prevention and control of seizures, mainly through the projection of fibers issued by the LC nuclei to the cerebral cortex and subcortical structures to prevent limbic system seizures [10].

Because epilepsy is a pathological state of brain functional connections, it is of great significance to study the mechanism of epilepsy from the perspective of the brain network [11]. In patients with epilepsy, the functional network topology was usually disrupted by abnormal synchronous discharges [12]. Recent research has found that VNS can affect the connection patterns of separation and integration in patients with RE to control their seizures [13]. Lanzone et al. found that the small-world index, global efficiency (GE), and betweenness centrality of the brain functional network in epileptic patients changed significantly when VNS was turned on. However, quantitative electroencephalography showed that band-power was not affected [14]. In addition to changing the network topology, VNS also affects the functional connectivity strength of brain networks to control seizures. Coa et al. found that responders treated with VNS had decreased functional connectivity in the gamma and delta bands and increased functional connectivity in the alpha band. However, the functional connectivity of non-responders did not show significant differences before and after treatment [15].

Based on the previous research results, we raise the question of whether VNS has an effect on the network topology of epileptic patients in addition to affecting functional connectivity. Therefore, the aim of this study was to explore the changes in functional connectivity and small-world metrics in patients with RE before and after VNS treatment by calculating weighted phase lag index (wPLI) and small-world metrics [16]. Currently, most of the research primarily focuses on analyzing interictal data, and there is limited study on ictal data. To address this gap, we screened and analyzed electroencephalogram (EEG) data during the ictal. The reason for selecting ictal data is that, compared to interictal periods, it can more accurately reflect the seizure onset zone and the degree of brain impact. EEG data from the seizure period are often used in intracranial EEG to locate the seizure onset area [17]. Current assessments of the efficacy of VNS rely on a reduction in the number of seizures during follow-up, but this is largely dependent on patient participation. The onset of epilepsy can also be affected by objective factors such as the environment [18]. The results of network analysis in patients with controlled seizures can provide objective criteria for evaluating the efficacy of VNS. Based on this, we designed the following experiment.

## 2 Methods

### 2.1 Patient selection

This study retrospectively collected EEG data from 17 patients with RE who received VNS (PINS, MODEL G112) treatment at the Fifth Affiliated Hospital of Zhengzhou University between March 2017 and March 2021. Ten patients (five males and five females with an average age of 19.23 ± 7.37 years) were selected as subjects in this study after strict screening of inclusion criteria. ASM remained unchanged in all patients before and after VNS treatment (as shown in Table 1).

**Table 1:** Patient demographic characteristics and VNS parameters

Patient	Age	Sex	History of epilepsy (year)	Frequency (Hz)	Pulse-width (μs)	Intensity (mA)	Impedance (Ω)
1	11	F	4	30	250	1.2	3,500
2	18	F	5	30	250	1.6	2,600
3	15	M	2	30	250	1.4	2,800
4	21	F	11	30	250	1.8	2,800
5	13	M	5	30	250	1.6	2,600
6	34	M	14	30	250	2.0	5,000
7	28	F	1	30	500	2.0	4,800
8	23	M	6	30	500	1.6	2,659
9	9	M	2	30	250	2.0	2,568
10	20	F	7	30	250	1.4	1,930

Abbreviations: M: male, F: female.

The inclusion criteria were as follows: (1) epileptic focus cannot be located or are located in important functional areas; (2) complete preoperative and postoperative EEG review records were obtained; and (3) 2 years after VNS, the number of seizures was reduced by more than 50% compared with that before VNS treatment. The exclusion criteria were as follows: (1) the presence of neurological disorders other than epilepsy; (2) the previous presence of a serious underlying disease (i.e., heart failure); and (3) other neuromodulation treatments were received. All patients were followed up for 2 years after VNS treatment, and the average number of seizures per month was recorded and compared with the number of seizures recorded before surgery (as shown in Table 2). The number of seizures in patients was reduced by an average of 64% 2 years after VNS treatment. The prognosis of Mchugh is graded as grade I or II.

**Table 2:** Follow-up data of patients

No.	Type	AED	EZ	Pre-VNS	Pos-VNS	Reduction (%)	McHugh class
1	GTCS	OXC + TOP	Bitemporal	10	2	80	IA
2	CPS	LMT + VA	Left temporal	10	4	60	IIB
3	CPS	LMT + TOP	Right temporal	23	5	78	IIB
4	GTCS	VA + CBZ	Bitemporal	19	3	84	IA
5	MAS	VA	Left frontal	5	2	60	IIA
6	SPS	VA + CBZ	Left temporal	10	1	90	IB
7	GTCS	LMT + OXC	Bifrontal	7	3	57	IIA
8	SPS	VA + CBZ	Right temporal	12	4	67	IIB
9	GTCS	LMT + CBZ	Bitemporal	13	6	54	IIB
10	MAS	CBZ + TOP	Right frontal	5	1	80	IA

Abbreviations: AED: antiepileptic drug; GTCS, generalized tonic-clonic seizures; MAS, myoclonic-atonic seizures; SPS, simple partial seizure; CPS, complex partial seizure; VA, valproic acid; TOP, topiramate; CBZ, carbamazepine; LMT, lamotrigine; OXC, oxazepine.

### 2.2 Methods of EEG data collection

Patients avoided central nervous stimulants for 1 week prior to data collection. During the EEG collection, the patients were left alone in a quiet room. EEG data were collected using the international 10–20 lead Nicolet system. The resistance of each recording electrode did not exceed 5 kΩ, the sampling rate was 500 Hz, and a binaural mastoid process was used as the reference electrode. To ensure the accuracy of data collection, each patient was recorded for 48 or 72 h. During the recording process, patients were subjected to eye-closing and hyperventilation tests, and flash stimulation or special stimulation induction tests were performed if necessary to ensure that EEG fragments of patients during seizures were collected for more than 2 min during the monitoring period. For the recorded EEG data, we selected the EEG data recorded by 19 electrodes (Fp1, Fp2, F3, F4, F7, F8, Fz, T3, T4, T5, T6, C3, C4, Cz, P3, P4, Pz, O1, O2) for analysis. EEG data collection methods were consistent for each participant before and after VNS treatment.

### 2.3 Data selection and preprocessing

The data collected from each patient were carefully screened by two experienced neurophysiologists to extract ictal episodes from the recorded EEG data. The selection process was based on EEG waveforms and patient video recordings, using the following data selection criteria: (1) clear observation of evident seizures in the video recordings; (2) presence of large-scale synchronous discharge patterns in the EEG waveforms; (3) stable muscle signals with no abnormal lead connections; and (4) patients were in an awake state during the seizures, and each seizure lasted for at least 2 s. As each patient’s EEG data were recorded for over 24 h and induced experiments were conducted, multiple segments of ictal events were recorded. To maintain data consistency, a uniform standard was applied for data extraction: For each seizure, only 2 s or multiples of 2 s of data were extracted to ensure the continuity of the data after segmentation. Ultimately, each patient’s data was limited to a duration of 2 min. The EEG segments (2 min) selected from the ten participants were combined to create 20-min segments. The EEG data after 2 years of VNS treatment were also processed in the same manner for data selection. The EEGLAB toolbox was used to preprocess the screened EEG data and select bandpass filtering ranging from 0.5 to 45 Hz [19]. Average reference and independent component analyses were used for noise reduction and artifact removal. Finally, the pre- and post-treated EEG segments were divided into 2-s segments, resulting in a total of 600 segments. For in-depth statistical comparisons of the differences in brain network characteristics, EEG signals were divided into delta (0.5–4 Hz), theta (4–8 Hz), alpha (8–13 Hz), beta (13–30 Hz), and gamma (30–45 Hz) bands according to different frequencies.

### 2.4 Construction of the functional network

It has been shown that the wPLI is highly sensitive in describing the synchronization of EEG time series and can avoid the effect of volume conduction [20]. Therefore, we used the wPLI to quantify the strength of the functional connections between nodes and construct functional brain networks. The specific calculation formula for the wPLI is as follows:

(2.1) \[{\mathrm{w}}{\mathrm{PLI}}=\frac{|E\{|\xi \{X\}{\mathrm{sgn}}(\xi \{X\})|\}|}{E(|\xi \{X\}|)},]\]

where *X* refers to the cross-spectrum of the two time series, and *ξ* represents the virtual portion of the cross-spectrum. The wPLI values are usually between 0 and 1, with 0 indicating no synchronization between the two time series and 1 indicating complete synchronization.

### 2.5 Calculation of small-world metrics

We used the Brain Connectivity Toolbox toolkit to compare and analyze differences in small-world metrics [21]. The node degree, clustering coefficient (CC), characteristic path length (CPL), and GE of the functional brain network are calculated, where the degree \[{D}_{i}]\] of node \[i]\] is defined as follows:

(2.2) \[{D}_{i}=\mathop{\sum }\limits_{j=1}^{N}{w}_{{ij}},]\]

where \[N]\] is the number of nodes in the network, and \[{w}_{{ij}}]\] is the connection strength between nodes \[i]\] and node \[j]\] . The average degree \[D]\] of the network is the average degree of \[N]\] nodes, and the average coupling strength of the edges in the network is described and defined as follows:

(2.3) \[D=\frac{1}{N}\mathop{\sum }\limits_{i=1}^{N}{D}_{i}.]\]

The CC is typically used to measure the local connectivity and clustering characteristics of a network. Quantization was performed by calculating the ratio between the number of actual existing connection edges between nodes adjacent to a given node \[i]\] and the number of maximum possible connection edges between adjacent nodes. The corresponding formula is as follows:

(2.4) \[{C}_{i}=\frac{2{e}_{i}}{{m}_{i}({m}_{i}\left-1)},]\]

where \[{m}_{i}]\] is the number of nodes adjacent to node *i*, *e_i_* is the number of actual connection edges between nodes \[{m}_{i}]\] and \[{m}_{i}({m}_{i}\left-1)/2]\] is the maximum number of possible connection edges.

The average CC of the network is the average CC of *N* nodes, which reflects the closeness of the connections among all the nodes of the network. The calculation formula is as follows:

(2.5) \[C=\frac{1}{N}\mathop{\sum }\limits_{i=1}^{N}{C}_{i}.]\]

The CPL is the average of all shortest path lengths between all pairs of nodes, which describes the global characteristics of the network. The formula for calculating the feature path length is

(2.6) \[L=\frac{1}{N(N\left-1)}\sum _{i\ne j}{l}_{{ij}},]\]

where \[N]\] is the number of all nodes in the network and \[{l}_{{ij}}]\] is the shortest path length between node \[i]\] and node \[j]\] .

GE is a global feature of a network used to measure the efficiency of information transmission within a network. The calculation formula is as follows:

(2.7) \[{E}_{{\mathrm{global}}}=\frac{1}{N(N\left-1)}\sum _{i\ne j}\frac{1}{{l}_{{ij}}}.]\]

### 2.6 Statistical analysis

Statistical tests were used to identify significant differences in brain networks between the pre-VNS and post-VNS states. First, an independent two-sample *t*-test was employed to compare the significant changes in wPLI values (inter-nodal connectivity) between all pairs of EEG channels before and after VNS treatment. Specifically, each state’s each segment could generate a 19 × 19 network, resulting in a total of 600 networks. We performed statistical tests on these 600 networks from both states and applied false discovery rate (FDR) correction for multiple comparisons between different brain regions in the wPLI matrices [22]. For each small-world metrics, a non-parametric rank-sum test was used for statistical analysis. Since it involved comparing small-world metrics between the two states, FDR correction was not required, and the significance level was set at 0.05.


**Ethical approval:** The research related to human use has been complied with all the relevant national regulations, institutional policies, and in accordance with the tenets of the Helsinki Declaration and has been approved by the authors’ institutional review board or equivalent committee. This study was approved by the Medical Ethics Committee of the Fifth Affiliated Hospital of Zhengzhou University (KY202027).
**Informed consent:** Informed consent has been obtained from all individuals included in this study.

## 3 Results

### 3.1 Changes in functional connectivity across the full-frequency band

**Figure 1:**
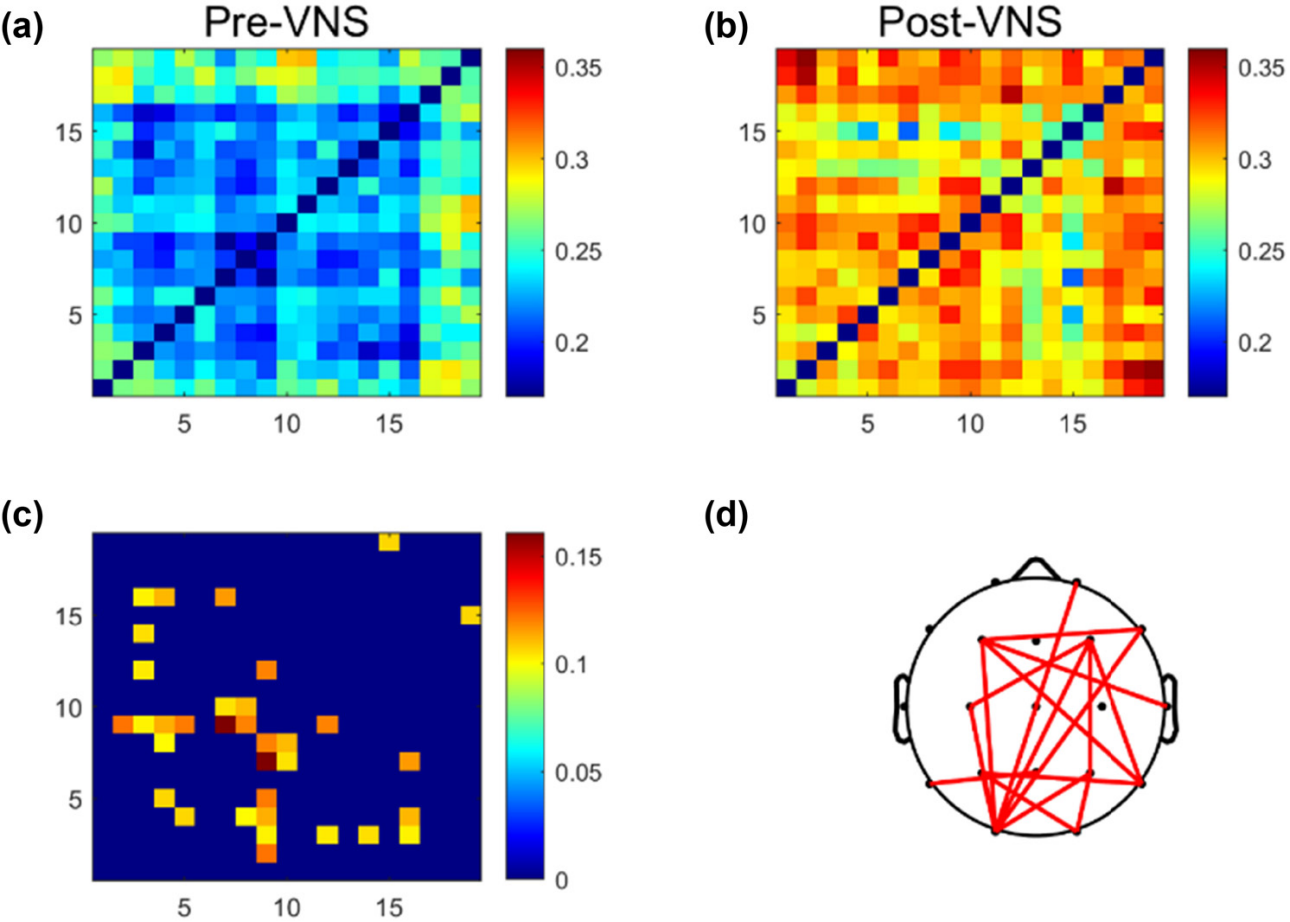
VNS-induced changes of the brain functional connectivity in the full-frequency band (0.5–45 Hz) EEG. (a) Brain correlation matrices before VNS treatment. (b) Brain correlation matrix after VNS treatment. (c) Functional connection matrices with significant differences. (d) Functional connections with differences were mapped to the brain topology.

First, we averaged the full-band wPLI of the 600 segments before and after VNS treatment to obtain functional connection matrices (as shown in Figure 1a and b). By performing independent two-sample *t*-tests and applying FDR correction to the changed wPLI, we obtained functional connections with significant differences (as shown in Figure 1). The functional connections with differences were then mapped to the brain topology (as shown in Figure 1d). We chose to use the top 10% of the fully connected brain network as the threshold to display the number of different brain network connections. The reason is that, after FDR correction, the remaining significant connections are fewer than 10% or less than 15%. Thus, we selected 10% as the threshold to ensure the presence of most connections while excluding weak connections. Red connections represent significantly enhanced functional connections after VNS treatment, while blue connections represent significantly decreased functional connections. Through statistical comparative analysis, we found that VNS treatment significantly enhanced the connection strength of certain brain regions, especially the functional connections in the parieto-occipital area (*P* < 0.01).

### 3.2 Changes in small-world metrics in the full-frequency band

**Figure 2:**
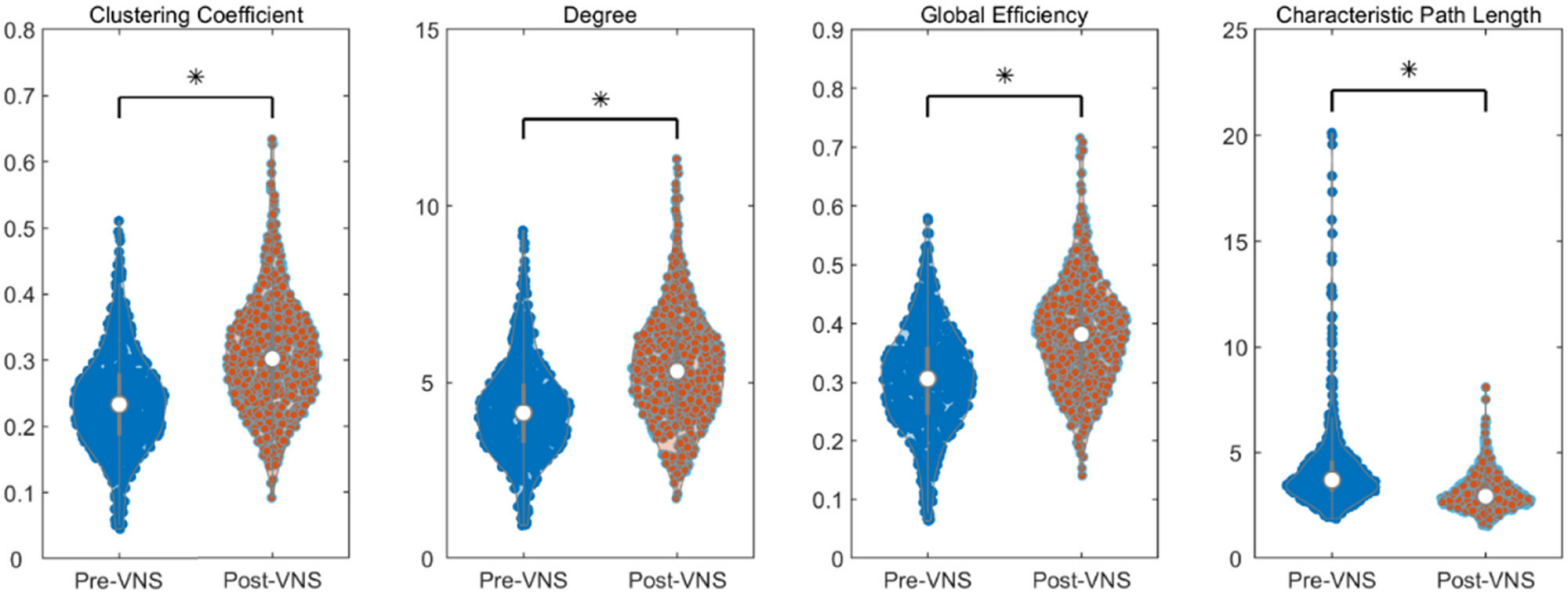
The change of small-world metrics in the full-frequency band (0.5–45 Hz), *P < 0.05.

Then, based on changes in brain network functional connectivity, we calculated the network properties in the full-frequency band (0.5–45 Hz) of EEG before and after VNS treatment, including average CC, average node degree, GE, and average shortest path length. The differences between 600 network properties before and after VNS surgery were assessed using the rank-sum test. The results showed that the CC, node degree, and GE of the brain network significantly increased after VNS treatment, while the average shortest path length significantly decreased (*P* < 0.01) (as shown in Figure 2). Each point in the figure represents the average value of a network, totaling 600 values. The gray boxes represent the 25th and 75th percentiles, and the white dots in the middle represent the median.

### 3.3 Functional connectivity changes in frequency sub-bands

**Figure 3:**
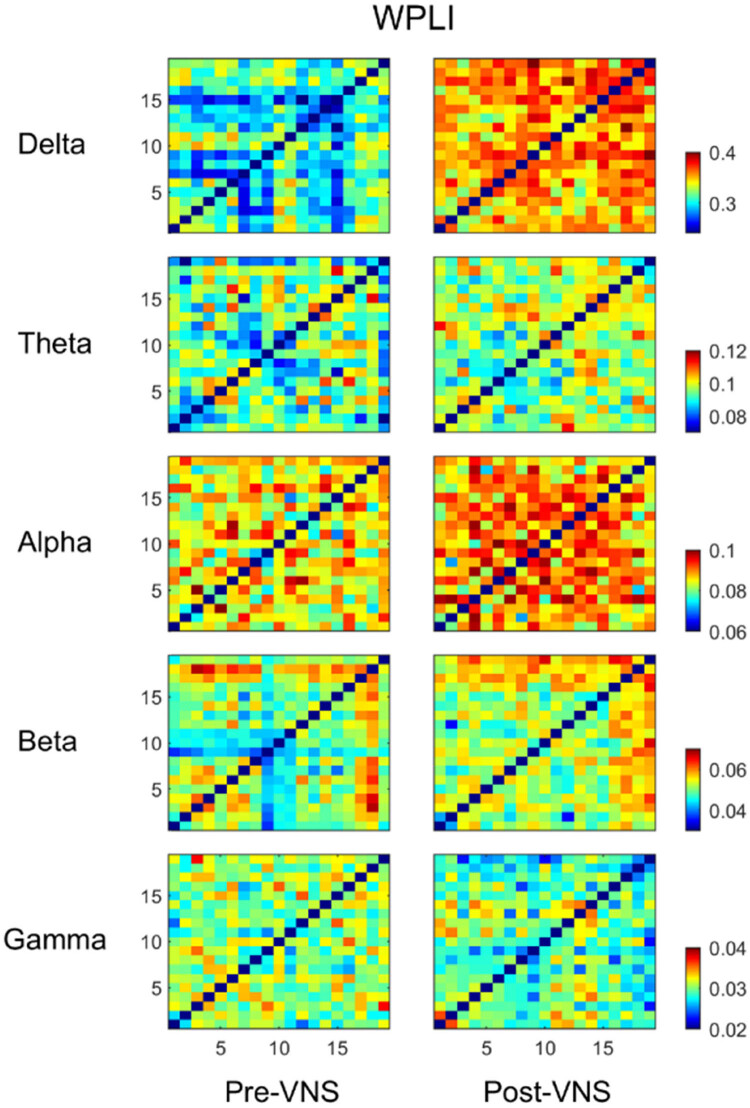
VNS-induced changes of the brain correlation matrices in five different frequency bands EEG.

We further explored the changes in the functional connectivity strength of brain networks corresponding to the delta, theta, alpha, beta, and gamma bands after VNS treatment. The average connection matrix corresponding to each frequency band was changed to varying degrees, especially the functional connection strength of the brain network in the delta and alpha bands, which was significantly different (*P* < 0.01) (as shown in Figure 3). These results indicated that the effects of VNS on the functional connectivity of brain networks are reflected in different frequency bands.

**Figure 4:**
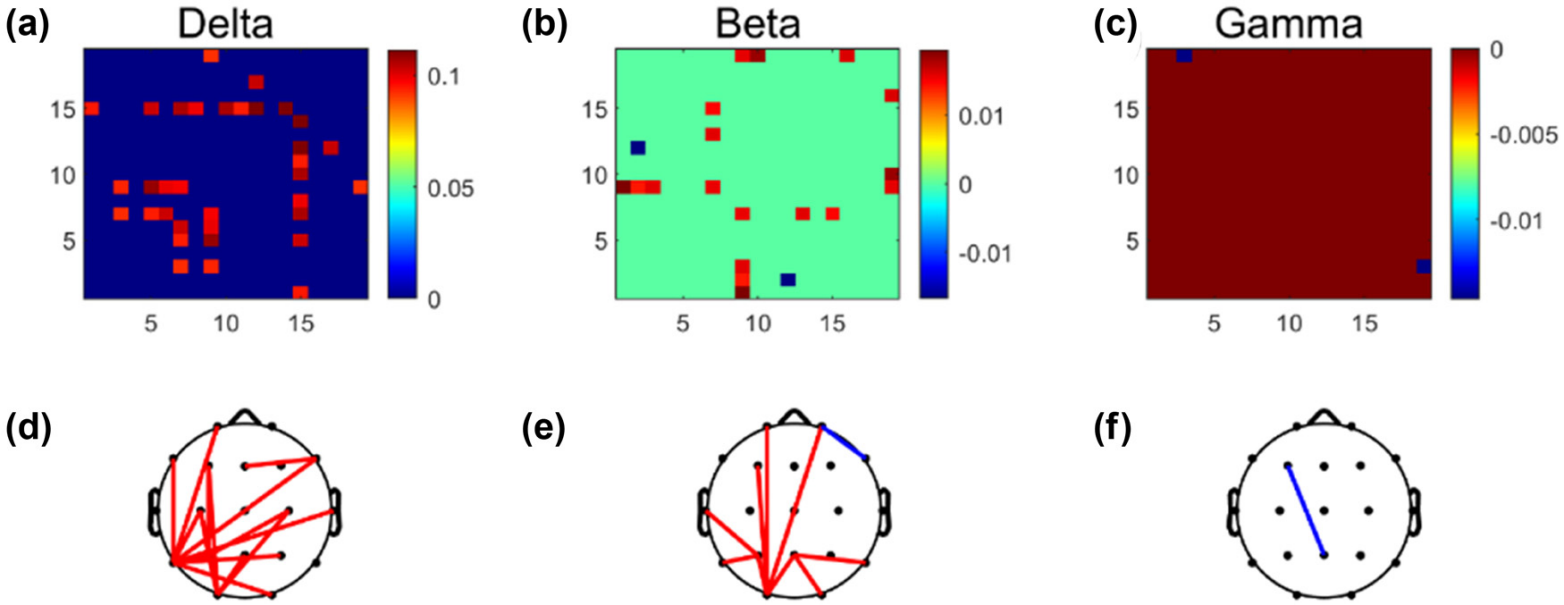
Brain functional connection matrices with significant differences in three different frequency bands of EEG: (a) delta band, (b) beta band, and (c) gamma band; (d)–(f) the brain network topology with significant differences in three frequency bands.

A two-sample *t*-test was used to compare the differences in wPLI corresponding to each frequency band before and after treatment (as shown in Figure 4). We found significant changes in the strength of connections in the functional brain networks in the delta, beta, and gamma bands after VNS treatment (*P* < 0.01). Among them, the wPLI corresponding to the delta and beta band was enhanced and gamma band was decreased. It is noteworthy that the significantly enhanced connections in the delta band functional brain network are closely related to the T5 and O1 nodes, whereas the enhanced connections in the beta band functional brain network are also connected to the O1 nodes. These results suggested that VNS therapy may specifically alter the connectivity strength of the functional brain networks in some frequency bands.

### 3.4 Changes in network properties in frequency sub-bands

**Figure 5:**
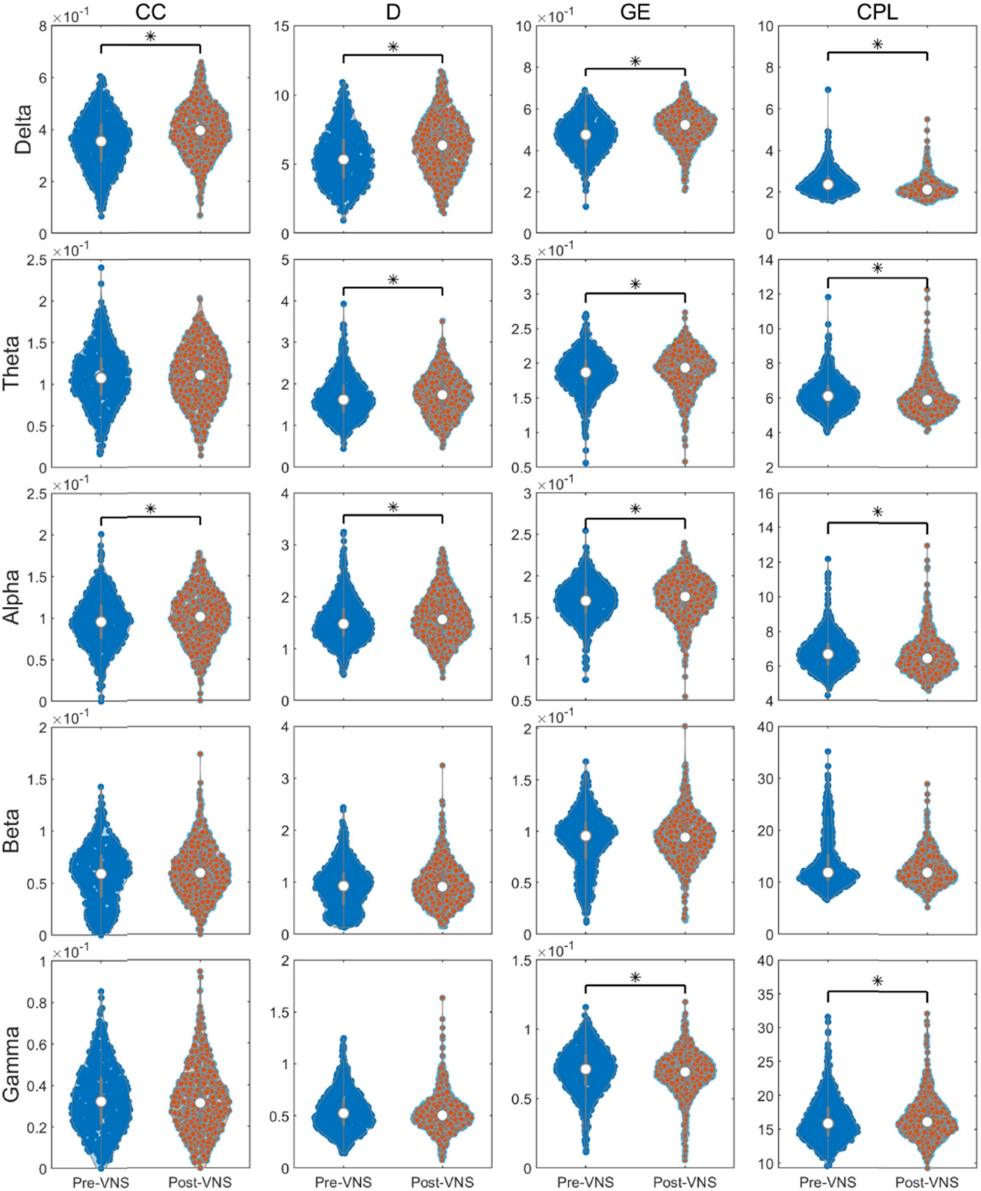
VNS-induced changes of the brain network characteristics in five different frequency bands EEG. *P < 0.05.

The small-world metrics of different frequency bands were calculated and statistically compared. The metrics of the delta and alpha band were significantly changed compared with those before treatment (*P* < 0.01), which showed that CC, D, and GE were increased and CPL was decreased (as shown in Figure 5). In the theta band, the D and GE were significantly increased, while CPL was significantly decreased (*P* < 0.01). However, GE decreased and CPL increased in the gamma band (*P* < 0.01). The results showed that after VNS treatment, reconfiguration of the brain network mainly occurred in the low-frequency band (<13 Hz). The changes in the brain network indices in the high-frequency band (>30 Hz) were opposite to those in the low-frequency band. Each point in the figure represents the average value of a network, totaling 600 values. The gray boxes represent the 25th and 75th percentiles, and the white dots in the middle represent the median.

## 4 Discussion

In this study, we evaluated the changes in brain networks induced by VNS by constructing the wPLI functional connectivity matrix and combining it with small-world network metrics. The results showed significant changes in functional connectivity and network properties in specific frequency bands after VNS treatment. Specifically, we observed increased functional connectivity between the frontal and parieto-occipital regions in the delta and beta bands, decreased functional connectivity in the gamma band, and weakened functional connectivity within the frontal region in the beta band. Overall, the network topology tended toward small-world characteristics in the entire frequency band after VNS treatment. However, further analysis showed that these changes predominantly occurred in specific frequency bands. Specifically, the small-world characteristics were significantly enhanced in the delta, theta, and alpha bands, while they were significantly reduced in the gamma band. No significant differences were observed in the beta band.

### 4.1 Relationship between VNS and brain network synchronization in ictal

In the analysis of the functional connectivity (FC) of the brain network, VNS increased the FC between the brain regions and tended to be more synchronous. By analyzing the wPLI of each frequency band, it was found that the wPLI mainly increased in the parieto-occipital regions of the delta band and beta band. However, the parieto-occipital area of the gamma band significantly decreased (*P* < 0.05). The emergence of epileptic discharges affected the activity pattern of neurons in the brain and led to hypersynchronous activity in different brain areas [23]. The antiepileptic effect of VNS mainly lies in EEG desynchronization, which is known to be associated with increased resistance to seizures [24].

Interestingly, our results were contrary to the previous literature conclusion that VNS has the effect of enhancing EEG synchronization. The most significant change of all frequency bands is in the delta band. The amount of wPLI enhancement between electrode pairs in the delta band is far greater than in other frequency bands. VNS achieves its goal of effectively controlling epileptic seizures by stimulating the vagus nerve and the related solitary nuclei (NTS) and locus coeruleus (LC), which in turn regulates the release of inhibitory neurotransmitters [25]. In the mouse model, it can be found that the number of spike waves on ictal EEG was significantly reduced after VNS implantation, and the spike firing was mainly caused by the highly oscillatory activity of neurons on the gamma band [26]. We speculated that patients treated with VNS have reduced synchronization of fast waves and increased synchronization of slow waves during episodes to reduce the number of sharp waves.

In addition, synchronization versus desynchronization during seizures was also a controversial issue [23]. Because the neuronal dynamics of the brain during the ictal are a complex process, the seizure onset area can be functionally organized into small neural components. In the process of EEG recording, if the recording electrode and the seizure area are not in the same spatial position, the phenomenon of asynchronism between the two may be detected in the early seizure. With the spread of abnormal discharge, the whole brain area will also tend to the level of synchronization and peak in the final stage of seizure. So there is a theory that when there is nothing left to ignite; epileptic seizures might stop when there is nothing left to excite [27]. According to our results, VNS increased the level of whole-brain synchronization throughout the seizure, which is consistent with the above idea. VNS promotes seizure termination by enhancing the level of global brain connectivity in RE patients during seizure.

### 4.2 The small-world metrics of the brain network changed

In the analysis of the brain functional network topology structure, we found that the change in the relationship between network segregation and integration in specific frequency bands. We draw a violin diagram based on the calculated small-world metrics of all networks. The dashed line values in the graph reflect changes in network properties under different states or conditions, not data for individual channels. Such graphs help to better understand the changes in overall network connectivity and the differences in small-world metrics across states [28].

The results showed that VNS can increase the transmission capacity of local and global information in delta and alpha bands. In the theta band, the global information transmission capability is improved. The changes in small-world metrics in these frequency bands all suggested that VNS significantly increased the small-world features of functional brain networks. However, in the gamma band, the global information transmission capability decreases, making the brain networks into a more regular network.

VNS has the same effect on the delta band and alpha band, which may be due to the increased activity of these two frequency bands during the ictal period, which improves the efficiency of information transmission between nodes. Since the delta band is an important neural marker for patients with epilepsy, we also found increased synchronization of the theta band in functional connectivity (wPLI) calculations. This indicated that VNS has the effect of promoting the propagation of slow wave bands during the ictal. However, the efficiency of global information transmission in the patient's functional network within the gamma band exhibited a significant reduction. Neuronal oscillatory activity in the gamma band is linked to the creation and propagation of epileptic discharges. Earlier research indicated that the decreased efficiency of information transmission within the gamma band's functional network correlates with a lower frequency of seizures [29]. We further confirmed that the gamma band also showed a decreasing trend of diffusion activity during the ictal.

The brain is a highly differentiated nervous system. Each brain region coordinates with another to complete physiological activities [30]. The brain networks of healthy individuals have normal integration and discrete information abilities. From the perspective of graph theory, the nodes are highly clustered. A small number of remote connections can improve the efficiency of information transmission and reduce transportation costs. But frequent abnormal epilepsy discharges in epileptic patients can disrupt this network structure and transform it into a more regular brain network (increased CC and CPL) [31]. The results also showed that VNS in the overall band can further improve the patients with functional brain networks of small-world properties, which may help explain why VNS can enhance memory and improve cognitive dysfunction caused by the seizure [25].

This study also had some limitations. First, we did not include a sufficient number of participants in the EEG data analysis, and this may not be able to make the results of the study have good representativeness. Therefore, we need to state here the limited scope of application. Second, a nonresponsive control group that could be referenced was not included, which would have affected the interpretation of the results for the mechanism of VNS. Therefore, further prospective studies with a large number of patients receiving VNS treatment and grouped according to their efficacy are needed to clarify the effect of VNS on functional network topology.

## 5 Conclusion

In this study, we established a functional connection matrix and brain network characteristic index to obtain the differences in brain network characteristics in RE patients before and after the VNS treatment. The seizures were effectively controlled in patients with RE. The brain network structure changed after VNS treatment. Changes in brain network characteristics and synchronous activities occur in specific frequency bands, and it has generally been shown that synchronization is enhanced and information transfer efficiency improves. These findings allow us to understand the underlying physiological mechanism of VNS in controlling epileptic seizures and help explore neurophysiological indicators of VNS efficacy.
